# Seborrheic Dermatitis and Dandruff: A Comprehensive Review

**DOI:** 10.13188/2373-1044.1000019

**Published:** 2015-12-15

**Authors:** Luis J. Borda, Tongyu C. Wikramanayake

**Affiliations:** Department of Dermatology and Cutaneous Surgery, University of Miami Miller School of Medicine, 1600 NW 10th Avenue, RMSB 2023A, Miami, Florida 33136, USA

**Keywords:** Seborrheic dermatitis, Dandruff, Sebaceous gland, *Malassezia*, Epidermal barrier

## Abstract

Seborrheic Dermatitis (SD) and dandruff are of a continuous spectrum of the same disease that affects the seborrheic areas of the body. Dandruff is restricted to the scalp, and involves itchy, flaking skin without visible inflammation. SD can affect the scalp as well as other seborrheic areas, and involves itchy and flaking or scaling skin, inflammation and pruritus. Various intrinsic and environmental factors, such as sebaceous secretions, skin surface fungal colonization, individual susceptibility, and interactions between these factors, all contribute to the pathogenesis of SD and dandruff. In this review, we summarize the current knowledge on SD and dandruff, including epidemiology, burden of disease, clinical presentations and diagnosis, treatment, genetic studies in humans and animal models, and predisposing factors. Genetic and biochemical studies and investigations in animal models provide further insight on the pathophysiology and strategies for better treatment.

## Introduction

Seborrheic Dermatitis (SD) and dandruff are common dermatological problems that affect the seborrheic areas of the body. They are considered the same basic condition sharing many features and responding to similar treatments, differing only in locality and severity. Dandruff is restricted to the scalp, and involves itchy, flaking skin without visible inflammation. SD affects the scalp as well as face, retro-auricular area, and the upper chest, causing flaking, scaling, inflammation and pruritus, and can have marked erythema. Flaking in SD and dandruff is usually white-to-yellowish, and may be oily or dry.

It is estimated that SD and dandruff combined affect half of the adult population. Despite such high prevalence, their etiology is not well understood. Various intrinsic and environmental factors, such as sebaceous secretions, skin surface fungal colonization, individual susceptibility, and interactions between these factors, all contribute to the pathogenesis. Genetic, biochemical studies and investigations in animal models further provided insight on the pathophysiology and strategies for better treatment. In this comprehensive review, we summarize the current knowledge on SD and dandruff, and attempt to provide directions for future investigations and treatments.

## Epidemiology

SD is a common dermatological disorder in the United States and worldwide [1]. Its incidence peaks during three age periods - in the first three months of life, during puberty, and in adulthood with an apex at 40 to 60 years of age [1–4]. In infants up to three months of age, SD involves the scalp (termed “cradle cap”), the face, and diaper area. Incidence can be up to 42% [4–6]. In adolescents and adults, SD affects the scalp and other seborrheic areas on the face, upper-chest, axillae, and inguinal folds [4,7,8]. Incidence is 1–3% of the general adult population [3,9]. Men are affected more frequently than women (3.0% vs. 2.6%) in all age groups, suggesting that SD may be associated with sex hormones such as androgens [1,3,8]. No apparent differences were observed in SD incidence between ethnic groups [3].

SD is more prevalent in immune-compromised patients such as HIV/AIDS patients [7,10], organ transplant recipients [11,12], and patients with lymphoma [13]. The incidence among HIV patients ranges from 30% to 83% [9,10]. Most cases of SD in HIV patients are diagnosed with CD4+ T lymphocyte counts between 200 and 500/mm3 [3,14,15], and decreased CD4+ counts are often associated with worse SD. Fewer cases of SD were reported when CD4+ T cells were more than 500/mm3 [14]. These observations suggest that immunological defects may play a role in SD.

SD is also associated with neurological disorders and psychiatric diseases, including Parkinson’s disease, neuroleptic induced parkinsonism, tardive dyskinesia, traumatic brain injury, epilepsy, facial nerve palsy, spinal cord injury and mood depression [4,5,16,17], chronic alcoholic pancreatitis, hepatitis C virus [18,19], and in patients with congenital disorders such as Down syndrome [20]. Furthermore, seborrhea-like dermatitis of the face may also develop in patients treated for psoriasis with psoralen and ultraviolet A (PUVA) therapy [21].

Comparing with SD, dandruff is much more common, and affects approximately 50% of the general adult population worldwide. It is also more prevalent in males than females [22,23]. Dandruff starts at puberty, reaches peak incidence and severity at the age of about 20 years, and becomes less prevalent among people over 50 [23]. Incidence varies between different ethnic groups: in a study in the U.S. and China, dandruff prevalence was 81–95% in African Americans, 66–82% in Caucasians, and 30–42% in Chinese [23].

## Burden of Disease

It is estimated that at least 50 million Americans suffer from dandruff, who spend $300 million annually on over-the-counter products to treat scalp itching and flaking [22]. Besides physical discomfort such as itching, dandruff is socially embarrassing and negatively impacts patients’ self-esteem [22].

While SD is much less prevalent, outpatient office visits alone cost $58 million in the United States in 2004, and $109 million were spent on prescription drugs [24]. Together with over-the-counter products and hospital services, the total direct costs of SD were estimated to be $179 million, plus another $51 million indirect costs in the form of lost work days [24]. In addition, because SD frequently occurs on the face and other visible areas, it has significant negative effects on patients’ quality of life (QOL) in the form of psychological distress or low self esteem; the willingness to pay for relief of the symptoms was $1.2 billion [24]. Furthermore, although the QOL impact in SD patients ranked lower than in patients with atopic or contact dermatitis, it was found to be higher than skin ulcers and solar radiation damage, and women, younger patients, and subjects with higher educational level were more affected [24].

## Clinical Presentation and Diagnosis

### Clinical presentations

The clinical presentations of SD and dandruff in children and adults are summarized in Table 1. SD often presents as well-delimited erythematous plaques with greasy-looking, yellowish scales of varying extents in regions rich in sebaceous glands, such as the scalp, the retro-auricular area, face (nasolabial folds, upper lip, eyelids and eyebrows), and the upper chest. Distribution of the lesions is generally symmetrical, and SD is neither contagious nor fatal. SD has a seasonal pattern, presenting more frequently during winter, and improving usually during summer [5,25,26]. Additionally, aggravation of SD has been associated with sleep deprivation and stress [7,27,28].

In infants, SD may present on the scalp, face, retro-auricular area, body folds, and trunk; rarely it may be generalized. Cradle cap is the most common clinical manifestation. SD in children is usually self-limited [3,15]. On the other hand, in adults, SD is a chronic or relapsing condition, featured by erythematous patches, with flaky, large, oily or dry scales in sebum-rich areas such as face (87.7%), scalp (70.3%), upper trunk (26.8%), lower extremities (2.3%), and upper extremities (1.3%) [5,7,29]. Pruritus is not an obligatory feature, but it is often present, mainly in scalp involvement [2]. The main complication is secondary bacterial infection, which increases the redness and exudate and local irritation [3,15].

In immune-suppressed patients, SD is often more extensive, intense, and refractory to treatment [3,26,30]. It is considered an early skin presentation of AIDS in both children and adults [14]. SD may also be a cutaneous sign of the immune reconstitution inflammatory syndrome in patients with highly active antiretroviral therapy (HAART) [31]. However, there have also been reports of SD regression with HAART [10].

### Differential diagnosis

The main differential diagnosis of SD and dandruff includes psoriasis, atopic dermatitis (mainly in the pediatric form of SD), tinea capitis, rosacea, and systemic lupus erythematous (SLE) [3,7,8] (Table 2). While psoriasis can affect similar locations as SD, typical lesions in psoriasis are thicker and present as plaques sharply limited with silvery white scales [8,32]. Lesions in atopic dermatitis usually do not appear until after 3 months of age, while lesions in SD usually appear earlier and rarely affect extensor areas. Tinea capitis, a highly contagious disease, typically shows scaly patches of scalp hair loss associated with “black dots”, which represent distal ends of broken hairs [33]. Conversely, SD is not associated with hair loss. Rosacea usually targets the malar areas on the face, sparing the nasolabial folds, and do not have scales; on the other hand, facial SD lesions are usually scaly, and affect the nasolabial folds, eyelids, and eyebrows, without associated flushing or telangiectasias [7,8,34]. Finally, skin lesions in SLE often follow a clear photo distribution, such as acute flares of bilateral malar rash, and may be associated with extra-cutaneous abnormalities such as arthritis, mouth ulcers, glomerulonephritis or cardiomyopathy [8,35]; SD does not have a photo distribution pattern, and does not affect organ systems other than the skin.

Other less common conditions that may resemble SD are pemphigus foliaceous, pityriasis rosea, secondary syphilis, diaper dermatitis and cutaneous Langerhans cell histiocytosis [3,4,7,30], which are summarized in Table 2. The majority of these conditions can be differentiated by clinical presentation and history; although syphilis, pemphigus foliaceous and SLE may require laboratory confirmation.

Additionally, some drugs (griseofulvin, ethionamide, buspirone, haloperidol, chlorpromazine, IL-2, interferon-α, methyldopa, psoralens) and nutritional deficiencies (pyridoxine, zinc, niacin and riboflavin) may induce an SD-like dermatitis, although the mechanism remains unknown [36,37]. These conditions can coexist with SD as well, making the diagnosis more challenging.

## Pathology

Diagnosis of SD is typically made by history and physical examination. In rare cases, a skin biopsy is needed for differential diagnosis. Histologically, the development of SD can be divided into two stages. In the acute and sub-acute stages, SD shows superficial perivascular and perifollicular inflammatory infiltrates, composed mainly of lymphocytes and histiocytes in association with spongiosis and psoriasiform hyperplasia, and can be coupled with parakeratosis around follicular opening (“shoulder parakeratosis”). Neutrophils can also be found in the scale crust at the margins of follicular ostia. On the other hand, in chronic lesions, marked psoriasiform hyperplasia and parakeratosis can be present with dilation of the venules of surface plexus which resembles psoriasis [3,4,38]. However, in psoriasis parakeratosis is often associated with thinning or loss of the granular layer due to accelerated keratinocyte differentiation.

Dandruff shows many common features as SD in histology, such as epidermal hyperplasia, parakeratosis, and *Malassezia* yeasts surrounding the parakeratotic cells [23]. Whereas inflammatory cells such as lymphocytes and NK cells may be present in great numbers in SD, dandruff shows subtle neutrophil infiltration or no infiltration. These findings support the notion that dandruff and SD are of a continuous spectrum of the same disease entity with different severity and location [39].

## Treatment

Treatment of SD and dandruff focuses on clearing signs of the disease; ameliorating associated symptoms, especially pruritus; and maintaining remission with long-term therapy. Because the main underlying pathogenic mechanisms involve *Malassezia* proliferation and local skin irritation and inflammation, the most common treatment is topical antifungal and anti-inflammatory agents (Table 3). Other widely used therapies are coal tar, lithium gluconate/ succinate and phototherapy (Table 3). New therapies have also emerged including immune modulators such as topical calcineurin inhibitors, and metronidazole, but their efficacy remains controversial [5]. Alternative therapies have been reported as well, such as tea tree oil [40,41]. Some factors to be considered before selecting a treatment include efficacy, side effects, ease of use/compliance, and age of the patient [5]. Systemic therapy is needed only in widespread lesions and in cases that do not respond to topical treatment [3,26].

## Pathophysiology

Despite the high prevalence, the pathogenesis of SD and dandruff is not well understood. However, studies have identified several predisposing factors, including fungal colonization, sebaceous gland activity, as well as several factors that confer individual susceptibility [2].

### Fungal colonization

Several lines of evidence suggest a pathogenic role for yeasts of the genus *Malassezia* in SD and dandruff [42–46]. *Malassezia* are lipophilic yeasts that are found mainly on seborrheic regions of the body [5,7,47]. Studies have detected *Malassezia* on the scalp of dandruff patients [45,48], and higher numbers of *Malassezia* (*M. globosa* and *M. restricta*) correlate with SD appearance/severity [4,49,50]. Additionally, among the multiple chemical entities that are effective in treating SD and dandruff, such as azoles, hydroxypyridones, allylamines, selenium and zinc, the sole common mechanism of action is antifungal activity [47–49]. Furthermore, *Malassezia* was shown to have lipase activity, which hydrolyzes human sebum triglycerides and releases unsaturated fatty acids such as oleic and arachidonic acid [51,52]. These metabolites cause aberrant keratinocytes differentiation, resulting in stratum corneum abnormalities such as parakeratosis, intracellular lipid droplets, and irregular corneocyte envelope [53]. Such changes lead to disrupted epidermal barrier function and trigger inflammatory response, with or without visible local inflammation. In addition, these metabolites induce keratinocytes to produce pro-inflammatory cytokines such as IL-1α, IL-6, IL-8 and TNF-α, thus prolonging the inflammatory response [39,54]. Furthermore, arachidonic acid can be a source of prostaglandins, which are pro-inflammatory mediators that can cause inflammation via neutrophil recruitment and vasodilation [38]. Interestingly, *Malassezia* infection has also been reported in goats, dogs and monkeys with seborrhea (dry or greasy) and dermatitis [55– 59].

While these observations support a pathogenic role for *Malassezia* in SD and dandruff, there is also strong evidence suggesting that individual predispositions and host interactions with *Malassezia*, rather than the mere presence of *Malassezia*, contribute to SD and dandruff pathogenesis. For example, *Malassezia* was detected on normal skin of majority of healthy adults, making it a commensal organism [2,5,26]. Moreover, while topical application of oleic acid did not induce visible changes in non-dandruff subjects, it caused skin flaking on the non-lesional scalp of dandruff patients [48]. These observations are suggestive of intrinsic epidermal barrier defects in the pathogenesis of SD and dandruff [48].

### Sebaceous gland activity

Sebaceous glands (SGs) are distributed over the entire skin surface in humans, except on the palms and soles. Secretion of sebum is highest on the scalp, face and chest [44]. Sebum production is under hormonal control, and SGs are activated at birth under the influence of maternal androgens via androgen receptors in sebocytes [60]. SGs are activated again at puberty under the control of circulating androgens [38,61], resulting in increased sebum secretion during adolescence, which is kept stable between 20 and 30 years of age and is then reduced [62]. During the period of active sebum secretion, the secretion rate is higher in males and stays high longer, between 30 and 60 years of age; in females, the rate drops fast after menopause [44]. Thus, SD and dandruff have a strong time correlation with SG activity, with cradle cap after birth, increased incidence throughout the teens, between third and sixth decades and then decreasing [3,4,9]. However, SD patients may have normal sebum production, and individuals with excessive sebum production sometimes don’t develop SD [38,63]. These findings suggest that while SG activity strongly correlates with SD and dandruff, sebum production by itself is not a decisive cause.

In addition to the level of sebum production, abnormalities of lipid composition may also play a role in SD development, likely through a favorable milieu for *Malassezia* growth [64]. In patients with SD, triglycerides and squalene were reduced, but free fatty acids and cholesterol were considerably elevated [38,44]. The elevated levels of free fatty acids and cholesterol may be the result of triglyceride degradation by *Malassezia’s* lipase, and these metabolites promote *Malassezia* growth and lead to recruitment of inflammatory infiltrates in the skin [64].

### Individual susceptibility

Besides sebaceous activity and *Malassezia* colonization, other factors also contribute to the pathogenesis of SD. Epidermal barrier integrity, host immune response, neurogenic factors and emotional stress, and nutritional factors have all been shown to play a role in individual susceptibility.

#### Epidermal barrier integrity

The stratum corneum (SC), the anucleated outer layers of the epidermis, functions as a barrier against water loss and entry of microorganisms and harmful agents from the environment [65]. The SC consists of several layers of terminally differentiated keratinocytes, the “corneocytes”, encased in lipid lamellae, held together by specialized intercellular cell adhesion structures called corneodesmosomes [66]. Any changes in the lamellar lipid composition, corneocyte size or shape, corneodesmosome number and SC thickness, could lead to alterations in the epidermal permeability barrier (EPB) function [66].

Normally, sebum may influence intercellular lipid organization to aid desquamation [66,67]. In SD and dandruff, however, altered corneodesmosomal hydrolysis may disrupt lipid organization and disturb the desquamation process, leading to aberrant barrier function [53,68]. In support of this notion, barrier structural abnormalities have been detected in dandruff scalp by electron microscopy that included intercellular *Malassezia* yeasts, changes in corneocyte shape and corneodesmosomes, and disrupted lipid lamellar structure [23,53,66]. Consistent with the structural findings, dandruff patients have been found to be more reactive (higher itch perception or flaking) than controls to topical applications of histamine or oleic acid to the scalp [48,69,70]. These observations indicate that disrupted EPB function can contribute to the aggravation of dandruff. Recent genetic studies in humans and animals suggest that disrupted barrier function may even directly cause SD-like conditions [71]. Biochemical analysis further demonstrated that dandruff skin displayed altered protein profiles as well as those of SC ceramides and free fatty acids, in the absence of apparent inflammation [72]. These studies underscore the importance of barrier restoration and maintenance in the management of SD and dandruff.

#### Immune response

Both the incidence and severity of SD are associated with immune-suppression, particularly in HIV/AIDS patients. Because no clear differences were found in *Malassezia* levels between individuals with and without SD in this population, it is likely that an immune or inflammatory reaction could be the predisposition [5,9]. Indeed, one study found elevated levels of human leukocyte antigens HLA-AW30, HLA-AW31, HLA-A32, HLA-B12 and HLA-B18 in SD [3,73,74]. Additionally, increased levels of total serum IgA and IgG antibodies have been detected in SD patients [75]. However, no increase in the titers of antibodies against *Malassezia* was detected, suggesting that the elevated immunoglobulin production occurs rather as a response to yeast metabolites [26,75,76]. The strong inflammatory reaction provoked by these metabolites includes infiltration of Natural Killer (NK) cells and macrophages, with concurrent local activation of complement and an increased local production of inflammatory cytokines, such as IL-1α, IL-1β, IL-6 and TNF-α in affected skin areas [54]. The lack of increase in anti-*Malassezia* antibodies also indicates a change in cellular immune response instead of humoral response [76,77]. The specific role of lymphocyte activity remains controversial [76–79].

#### Genetic factors

The genetic components of SD and dandruff had been under-appreciated until recently, when studies in animal models and humans identified inherited dominant and recessive forms of SD and dandruff. In the autosomal recessive “inherited seborrheic dermatitis” (seb) mice, a spontaneous mutation in the outbred Him:OF1 mice caused seborrhea, rough coat, alopecia, growth retardation, and sometimes abnormal pigmentation in homozygous mutants [80]. Histological examination revealed enlarged sebaceous glands, hyperkeratosis, parakeratosis, acanthosis and inflammatory infiltrates in the epidermis and dermis. Neither yeasts nor dermatophytes were detected. These mice were the first animal model of SD to show a clear mode of inheritance, though the underlying mutation remains unidentified [80,81].

Consistent with a role for altered immunity in the pathogenesis of SD, transgenic mice carrying the 2C T cell receptor (TCR) transgene in the DBA/2 background developed extremely inflammatory phenotype in seborrheic areas, such as the ears, around the eyes, and muzzle area [82]. Additionally, positive fungal staining by PAS was consistently detected in lesional skin but not readily apparent in non-lesional skin from diseased mice or from DBA/2 control mice. Furthermore, antifungal treatment reversed clinical and pathology presentations, and reduced PAS staining [82]. These observations support the notion that immune compromise and fungal infection play active roles in SD.

Another spontaneous mutant mouse strain that shows SD-like phenotype is the rough coat (*rc*) mice, which showed sebaceous hypertrophy and greasy hair coat, alopecia, and growth retardation [83]. The *rc* is transmitted in an autosomal recessive mode. We have since identified the cause of the *rc* phenotype to be a missense mutation in the *Mpzl3* gene, which is expressed in the superficial layers of the epidermis [84,85]. Our *Mpzl3* knockout mice recapitulated the *rc* phenotype, and mice with white hair coat developed more severe and persistent inflammatory skin phenotype and dandruff in the seborrheic areas [85]. We have shown that the early-onset inflammatory skin phenotype was not caused by immune defects [85]. However, skin abnormalities in *Mpzl3* knockout mice and perturbed epidermal differentiation in organotypic human skin models with *MPZL3* knockdown indicate that *MPZL3* is a key regulator of epidermal differentiation [85,86]. Interestingly, a frame-shift mutation in ZNF750, a transcription factor controlling epidermal differentiation and an upstream regulator of *MPZL3*, caused autosomal dominant seborrhea-like dermatitis in patients [71,86]. These studies in humans and animal models underscore the consequence of abnormal epidermal differentiation in the pathogenesis of SD and dandruff, and have provided the genetic basis for some of the predisposing factors discussed above. These animal models will be important tools to dissect the underlying pathways that will identify novel targets for better treatment of these disorders.

#### Neurogenic factors and emotional stress

The high incidence of SD in patients with Parkinson’s disease [17,87,88] and neuroleptic-induced Parkinsonism [89,90] has long been observed, especially in those with severe seborrhea, which provides favorable conditions for *Malassezia* proliferation. Bilateral seborrhea has been observed in patients with unilateral Parkinsonism, suggesting that these sebum changes were likely regulated neuro-endocrinologically rather than purely neurologically [5,26,91]. Consistent with this notion, α-melanocyte stimulating hormone (α-MSH) levels were elevated in Parkinson patients, possibly due to inadequate dopaminergic input. Moreover, treatment with L-dopa reduced α-MSH, and re-established the synthesis of MSH-inhibiting factor, reducing sebum secretion [26,92].

Additionally, there is evidence for a link between neurological damage (e.g. traumatic brain, spinal cord injury) and SD [93]. Facial immobility of Parkinsonian patients (mask-like face) and immobility due to facial paralysis can induce elevated sebum accumulation and lead to SD, but only on the affected side [26,43,94]. Because poor hygiene has been implicated in SD, these observations suggest that sustained reservoirs of residual sebum associated with immobility may influence the manifestation of the disease [3,22,26,88]. SD is also more commonly seen in depressive disorders and emotional stress [5,16].

#### Other factors

In the past, nutrition has been studied as a possible contributing factor for SD. Zinc deficiency in patients with acrodermatitis enteropatica, riboflavin, pyridoxine and niacin deficiency can manifest seborrheicdermatitis-like rash [26,36]. Other medical conditions, such as familial amyloidotic polyneuropathy and Down syndrome, have also been associated with SD [95,96].

**Figure 1:**
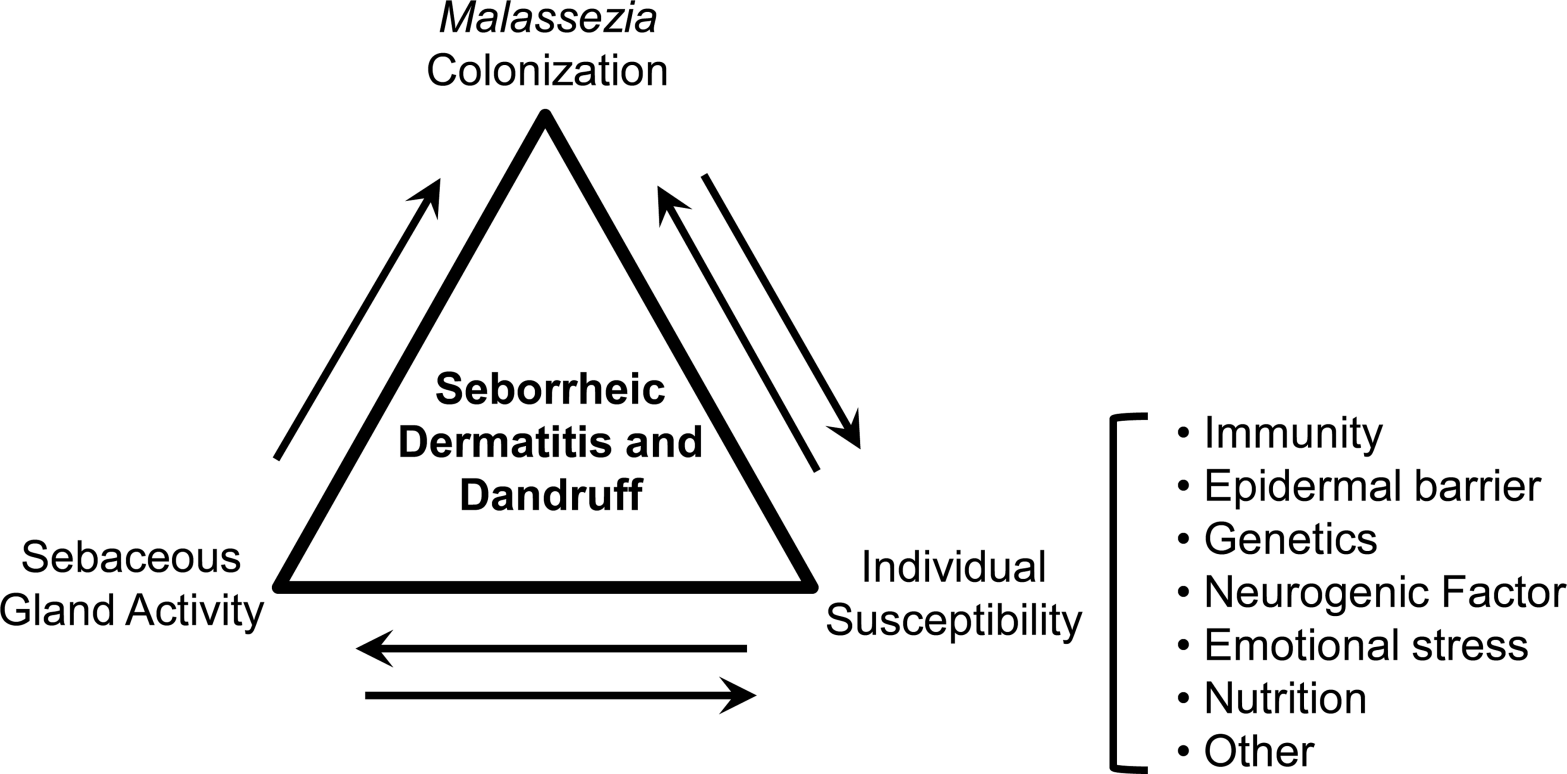
Predisposing factors and their interactions in the pathogenesis of seborrheic dermatitis and dandruff.

In summary, multiple predisposing factors have been identified in the pathogenesis of SD and dandruff (Figure 1). The presence and abundance of *Malassezia* yeast, host epidermal conditions and sebaceous secretion, combined with various other factors, and interactions between these factors, determine an individual’s susceptibility to SD and dandruff. In a likely scenario, there may be aberrant epidermal barrier function due to genetic predisposition, and excessive or altered sebum composition would exacerbate EPB disruption and provides a favorable milieu for *Malassezia* colonization. Disrupted EPB function facilitates entry of *Malassezia* and its metabolites, and irritates the epidermis and elicits host’s immune response. The host inflammatory response further disturbs epidermal differentiation and barrier formation, and pruritus and subsequent scratching would damage the barrier even further, leading to cycles of immune stimulation, abnormal epidermal differentiation, and barrier disruption.

## Conclusions

SD and dandruff are of a continuous spectrum of the same disease that affects the seborrheic areas of the body (Table 4). They share many common features and respond to similar treatments. Various intrinsic and environmental factors, such as *Malassezia* yeast, host epidermal conditions, sebaceous secretion, immune response, and the interactions between these factors, may all contribute to the pathogenesis. Effective management of SD and dandruff requires clearing of symptoms with antifungal and anti-inflammatory treatment, ameliorating associated symptoms such as pruritus, and general scalp and skin health to help maintain remission. Studies in humans and animal models to investigate the genetic and biochemical pathways will help identify new targets for the development of more efficacious treatment with less side effects, and better management of these conditions.

## Figures and Tables

**Table 1:** Clinical presentations of seborrheic dermatitis (SD) and dandruff.

		Features
**Dandruff**		Light, white to yellow and dispersed flaking on the scalp and hair without erythema. Absent to mild pruritus. Can spread to hairline, retro-auricular area and eyebrows.
**SD in Infants**	Scalp	Cradle Cap: Most Common. Red-yellow plaques coated by thick, greasy scales on vertex, appearing within 3 months of age.
	Face/Retro-auricular area	Erythematous, flaky, salmon-colored plaques on forehead, eyebrows, eyelids, nasolabial folds, or retro-auricular areas.
	Body folds	Lesions have moist, shiny, non-scaly aspects that tend to coalesce on neck, axillae or inguinal area.
	Trunk	More extensive form: Sharply limited plaques of erythema and scaling that cover lower abdomen.
	Generalized	Leiner’s Disease: Unusual, associated with immunodeficiency. Absent to mild pruritus. Concurrent diarrhea and failure to thrive. Spontaneous clearing within weeks to few months.
**SD in Adults**	Scalp	From mild desquamation to honey-colored crusts attached to scalp and hair leading to alopecia. May reach into forehead as scaly erythematous border known as “corona seborrheica”.
	Face/Retro-auricular area	Forehead, eyebrows, glabella or nasolabial folds. May spread to malar regions and cheeks in butterfly distribution.
		Eyelids: Yellowish scaling between eye lashes. Can lead to blepharitis with honey-colored crusts on free margin.
		Retro-auricular area: Crusting, oozing and fissures. May expand to external canal, with marked itching on occasionally secondary infection (otitis externa).
	Upper Chest	Petaloid type (common): small, reddish follicular and peri-follicular papules with oily scales at onset that become patches resembling a medallion (flower petals).
		Pityriasiform type: Widespread 5–15 mm oval-shaped, scaly macules and patches. Distributed along the skin tension lines (similar to extensive pityriasis rosea). New eruptions can continue for >3 months. Commonly on face and intertriginous areas.
	Body Folds	Moist, macerated appearance with erythema at the base and periphery on axillae, umbilicus, breast fold, genital or inguinal area. May progress to fissures and secondary infection.
SD with immune-suppression^*^		Extensive, severe and refractory to treatment. In both children and adults with AIDS†. Unusual sites involved such as extremities. More widespread with CD4 counts <200 cells/mm^3^. Associated with rosacea, psoriasis and acne.

* Human Immune-deficiency Virus (HIV), lymphoma and organ transplant recipients.

† AIDS: Acquired Immune-Deficiency Syndrome.

**Table 2:** Differential diagnosis of seborrheic dermatitis and dandruff.

Diagnosis		Diagnostic Clues
**Psoriasis**		Usually involves extensor, palmar, plantar, nails and extensor areas. Thick plaques sharply limited with silvery white scales. Positive family history. Arthritis present in 10% of patients. Uncommon in children.
**Atopic Dermatitis**		First appearance after 3 months of age, pruritus and restlessness are common. Frequently involves scalp, cheeks and extensor areas. Flexures involvement is more frequent in older ages. Family history of atopy such as eczema, allergic rhinitis and asthma. Self resolved by age 12.
**Tinea Capitis**		Commonly seen in children, frequently accompanied by hair loss patches with “black dots” (broken hair). Highly contagious. KOH examination of the hair shaft and fungal culture confirm the diagnosis. Household members of patient should be examined.
**Rosacea**		Usually targets the face. Papulopustules and telangiectasias on the malar, nose and perioral regions with slight desquamation. Recurrent edema and flushing.
**Systemic Lupus Erythematous (SLE)**		In acute stage, butterfly rash on face that spares the nose bridge or nasolabial folds. Photosensitivity is common. Skin lesions are generally associated with other clinical signs of SLE. Histology and serologic tests such as antinuclear autoantibodies confirm the diagnosis.
**Others**	Pemphigus Foliaceous	Erythema, scaling and crusting that first present on the scalp and face can expand to chest and back. Histology, direct immunofluorescence with anti-desmoglein antibodies confirm diagnosis.
	Pityriasis Rosea	Abrupt onset, appearance of herald patch and resolution within weeks.
	Secondary syphilis	Peripheral lymph-adenopathy, mucosal lesions and palmoplantar macula-papules. Serology tests such as VDRL/ RPR, FTA-ABS* confirm diagnosis.
	Diaper Dermatitis	Occurs on convex skin surfaces in contact with diaper, such as lower abdomen, genitalia, buttocks and upper thighs. Spares skin folds. Pustules are common.
	Langerhans cell histiocytosis	Multisystem disease. Brown to purplish papules prone to coalesce on the scalp, retro-auricular areas, axillae and inguinal folds. Possible lytic bone lesions, liver, spleen and lung involvement. Histology confirms diagnosis.

* VDRL: Venereal Disease Research Laboratory; RPR: Rapid Plasma Regain; FTA-ABS: Fluorescent Treponemal Antibody-Absorption.

**Table 3:** Treatment of seborrheic dermatitis and dandruff.

Medication			Dose/ Formulation	Regimen	Mechanisms	Side Effects	References
**TOPICAL**	Antifungals	Ketoconazole	2% Shampoo, cream, gel or foam	Scalp or skin: Twice/week × 4 weeks, then once/week for maintenance.	Inhibition of fungal cell wall synthesis.	ICD† in <1% of patients. Itching, burning sensation and dryness in 3% of patients.	[2,8,26,97–101]
		Bifonazole	1% shampoo, cream or ointment	Scalp: every other day or once daily. Skin: once daily.		ICD in 10% of patients.	[8,26,99,102]
		Miconazole	Cream	Skin: 1–2 times daily.		ICD, itching, burning sensation.	[47,97,103]
		Ciclopirox Olamine	1.5% shampoo, cream, gel or lotion	Scalp: 2–3 times/week × 4 weeks, then once/week for maintenance. Skin: twice daily.	Inhibition of metal-dependent enzymes.	ICD in <1% of patients. Itching, burning sensation in 2% of patients.	[8,47,97,99,104,105]
		Selenium sulfide	2.5% shampoo	Scalp: Twice/week × 2 weeks, then once/week × 2 weeks. Repeat after 4–6 weeks.	Cytostatic and keratolytic.	ICD in ~3% of patients. Orange-brown scalp discoloration.	[8,97,106,107]
		Zinc Pyrithione	1% shampoo	Scalp: 2–3 times/week.	Increased cellular copper interferes with iron-sulfur proteins.	ICD in ~3% of patients.	[8,97,99,101,108,109]
	Cortico-steroids	Hydrocortisone	1% cream	Skin: 1–2 times daily.	Anti-inflammatory, anti-irritant.	Risk of skin atrophy, telangiectasias, folliculitis, hypertrichosis, and hypopigmentation with prolonged use.	[8,9,97,99,103,108]
		Betamethasone dipropionate	0.05% lotion	Scalp and skin: 1–2 times daily.			[8,47,110]
		Desonide	0.05% lotion, gel	Scalp and skin: 2 times daily.			[8,111–113]
		Fluocinolone	0.01% shampoo, lotion or cream	Scalp or skin: Once or twice daily.			[7,114]
	Immuno-modulators	Pimecrolimus	1% cream	Skin: 1–2 times daily.	Inhibition of cytokine production by T-lymphocyte.	Risk of skin malignancy and lymphoma with prolonged use.	[47,98,115–118]
		Tacrolimus	0.1% ointment	Skin: 1–2 times daily × 4 weeks, then twice/week for maintenance.			[26,97,109,118–120]
	Miscellaneous	Coal tar	4% shampoo	Scalp: 1–2 times/week.	Antifungal, anti-inflammatory, keratolytic, reduces sebum production.	Local folliculitis, ICD on fingers, psoriasis aggravation, skin atrophy, telangiectasias, hyper-pigmentation. Risk of squamous cell carcinoma with prolonged use.	[4,8,47,117,121]
		Lithium gluconate/succinate	8% ointment or gel	Skin: twice daily × 8 weeks.	Anti-inflammatory via increased IL-10 and decreased TLR2 and TLR4 in keratinocytes.	ICD in <10% of patients.	[8,122–124]
		Metronidazole	0.75% gel	Skin: twice daily × 4 weeks.	Anti-inflammatory via inhibition of free radical species.	Rare contact sensitization with prolonged use.	[5,47,125,126]
		Phototherapy	UVB: Cumulative dose of 9.8 J/cm2	Three time/week × 8 weeks or until clearing.	Immuno-modulation and inhibition of cell proliferation.	Burning, itching sensation during/after therapy. Risk of genital tumor with prolonged use.	[26,127–129]
**SYSTEMIC**		Itraconazole	Oral: 200 mg	Once daily × 7 days, then once daily × 2 days/month for maintenance.	Inhibition of fungal cell wall synthesis. Anti-inflammatory via inhibition of 5-lipoxygenase metabolites.	Rare liver toxicity.	[97,130,131]
		Terbinafine	Oral: 250 mg	Once daily × 4–6 weeks or 12 days monthly × 3 months.	Inhibition of cell membrane and cell wall synthesis.	Rare tachycardia and insomnia.	[132–134]

**Note:** Shampoos, foams and lotions are better suited for treating seborrheic dermatitis and dandruff on the scalp; gels, creams and ointments are used to treat seborrheic dermatitis on body locations other than the scalp.

† ICD: Irritant Contact Dermatitis.

**Table 4:** Comparison of seborrheic dermatitis and dandruff.

	Seborrheic Dermatitis	Dandruff	References
**Epidemiology**	Up to 40% of infants within 3 months of age, 1–3% of the general adult population.	50% of adult population.	[1–3,22,23]
**Location**	Scalp, retro-auricular area, face (nasolabial folds, upper lip, eyelids, eyebrows), upper chest.	Scalp.	[2,7,15]
**Presentation**	Erythematous patches, with large, oily or dry scales.	White to yellow flakes dispersed on the scalp and hair; without erythema.	[2,3,26]
**Histology**	Acanthosis, hyperkeratosis, spongiosis, parakeratosis, *Malassezia* yeasts.		[3,23,38]
	Vasodilation and perivascular and perifollicular inflammatory infiltration; “shoulder parakeratosis”.	Subtle neutrophil infiltration or no inflammatory infiltration.	
**Treatment**	Antifungal shampoos and topical.		[2,8,26,47,97]
	Topical corticosteroids, immune modulators, phototherapy, systemic treatment.		
**Predisposing Factors and causes**	Sebaceous gland activity, fungal colonization, and individual susceptibility (epidermal barrier integrity, host immune response, genetic factors, neurogenic factors and stress, nutrition, etc.).		[2,3,9,15,26,44,66]

## Abbreviations

AIDS: Acquired Immune-Deficiency Syndrome
FTA-ABS: Fluorescent Treponemal Antibody-Absorption
HAART: Highly Active Antiretroviral Therapy
HIV: Human Immune-Deficiency Virus
ICD: Irritant Contact Dermatitis
QOL: Quality of Life
RPR: Rapid Plasma Regain
SC: Stratum Corneum
SD: Seborrheic Dermatitis
VDRL: Venereal Disease Research Laboratory
